# A Theory-Based Exercise App to Enhance Exercise Adherence: A Pilot Study

**DOI:** 10.2196/mhealth.4997

**Published:** 2016-06-15

**Authors:** Elizabeth C Voth, Nelly D Oelke, Mary E Jung

**Affiliations:** ^1^ School of Health and Exercise Sciences Faculty of Health and Social Development University of British Columbia: Okanagan Kelowna, BC Canada; ^2^ School of Nursing Faculty of Health and Social Development University of British Columbia: Okanagan Kelowna, BC Canada

**Keywords:** exercise, mHealth, app, self-monitoring, behavior change, social cognitive theory

## Abstract

**Background:**

Use of mobile health (mHealth) technology is on an exponential rise. mHealth apps have the capability to reach a large number of individuals, but until now have lacked the integration of evidence-based theoretical constructs to increase exercise behavior in users.

**Objective:**

The purpose of this study was to assess the effectiveness of a theory-based, self-monitoring app on exercise and self-monitoring behavior over 8 weeks.

**Methods:**

A total of 56 adults (mean age 40 years, SD 13) were randomly assigned to either receive the mHealth app (experimental; n=28) or not to receive the app (control; n=28). All participants engaged in an exercise goal-setting session at baseline. Experimental condition participants received weekly short message service (SMS) text messages grounded in social cognitive theory and were encouraged to self-monitor exercise bouts on the app on a daily basis. Exercise behavior, frequency of self-monitoring exercise behavior, self-efficacy to self-monitor, and self-management of exercise behavior were collected at baseline and at postintervention.

**Results:**

Engagement in exercise bouts was greater in the experimental condition (mean 7.24, SD 3.40) as compared to the control condition (mean 4.74, SD 3.70, *P*=.03, d=0.70) at week 8 postintervention. Frequency of self-monitoring increased significantly over the 8-week investigation between the experimental and control conditions (*P*<.001, partial η^2^=.599), with participants in the experimental condition self-monitoring significantly more at postintervention (mean 6.00, SD 0.93) in comparison to those in the control condition (mean 1.95, SD 2.58, *P*<.001, d=2.10). Self-efficacy to self-monitor and perceived self-management of exercise behavior were unaffected by this intervention.

**Conclusions:**

The successful integration of social cognitive theory into an mHealth exercise self-monitoring app provides support for future research to feasibly integrate theoretical constructs into existing exercise apps. In addition, findings provide preliminary support for theory-based apps to increase self-monitoring and exercise behavior in comparison to a control, no-app condition.

## Introduction

### Benefits of Exercise

The benefits of exercise are irrefutable [1] and have been demonstrated for individuals of all ages [2]. In Canada, the government encourages engagement in exercise through the publication of the Canadian Physical Activity and Sedentary Behaviour Guidelines Handbook [3]. While it is often assumed that knowledge of the benefits of exercise will increase exercise levels, informational campaigns have been rendered as ineffective for sustaining behavior change [4,5]. This is exemplified by the steady decline in physical activity and fitness levels among Canadians [2] despite the promotion of these physical activity guidelines, with only 15% of Canadian adults currently meeting daily physical activity recommendations [6]. Similar levels of engagement are seen within the United States [7] and Australia [8], with less than 50% of the respective populations engaging in adequate levels of physical activity.

### Mobile Health (mHealth) Technology

One strategy that may provide an effective medium to target physical inactivity at the population level is mobile health technology. To date, there are nearly 7 billion mobile phone subscriptions worldwide, with the use of mobile devices reaching 90% in developing countries and 96% globally [9]. This widespread use of mobile devices has led to the creation of mobile health-based (mHealth) products. Simultaneously, advances in technology have shifted traditional means of health promotion materials from read-only (eg, pamphlets and websites with read-only content) to interactive and responsive means (eg, mobile apps). mHealth apps offer many advantages over traditional informational materials. Data from users can now be analyzed in a context-appropriate, timely, and sophisticated manner [7]. More than ever before, there is opportunity to provide real-time support to the masses, outside of costly traditional personal training or counseling appointment times.

As of 2012, 84% of mobile phone owners had downloaded at least one app to their phone; 19% of those individuals had downloaded an app specifically related to tracking or managing a health-related behavior[10]. The continually increasing prevalence of app use further demonstrates the potential reach for mHealth exercise interventions. While app use is increasing in popularity, existing apps are not without limitations.

### Use of Evidence-Based Strategies

The majority of health-related apps currently available are developed on the traditional dissemination model. Generic, automated text messages are typically sent to individual users on a standardized time of day or week, or access to content is on a dedicated website in which users are referred to go read. While these apps provide valuable information to the user, such apps have neglected to integrate evidence-based strategies from established health behavior change theories [7,11-13]. In a 2012 review of the *Health and Fitness* category in the Apple App Store, Cowan and colleagues [11] concluded that there was an overarching lack of theoretical constructs used within 127 surveyed apps. Similar findings were reported in Direito and colleagues’ 2014 study evaluating the presence of 26 behavior change techniques in the 40 most popular physical activity and dietary apps from the Apple App Store [14]. While some incorporation of behavior change techniques is evident, Direito and colleagues' conclusions remained consistent with Cowan’s review, in that an absence of behavior change strategies exists in physical activity and dietary apps [14]. Cowan, Direito, and colleagues are not alone. Several other reports have also strongly recommended that future apps in the health domain be improved by incorporating evidence-based practices that are known to enhance health behavior change [11,15-17]. These findings highlight the need for collaboration between health behavior change experts and app developers.

Although rare to find, research-derived programs such as the Heart Exercise And Remote Technologies (HEART) mobile phone trial [18] demonstrate the benefits of integrating evidence-based behavior change strategies. Having established utilizing principles of behavior change from social cognitive theory [19], HEART uses a personalized, automated package of text messages aimed at increasing levels of exercise behavior in individuals with ischemic heart disease. HEART short message service(SMS) texts were developed to assist users with goal setting, exercise scheduling, and self-efficacy to overcome exercise barriers and engage in regular exercise in a positive and cost-effective manner.

As demonstrated in the HEART trial, social cognitive theory is particularly well suited for mHealth interventions, as the tenets of the theory are grounded in (1) self-monitoring, (2) self-evaluation, and (3) modification of current behavior based on this self-reflection [20,21]—tasks that mHealth apps have the capacity to assist the user with. The majority of apps allow the user to record their exercise sessions as a form of self-monitoring. Relatedly, many apps allow the user to look back at past exercise sessions in a summative format (eg, number of sessions completed last week)—an opportunity for self-evaluation and reflection. A main tenet within social cognitive theory is self-efficacy. Self-efficacy is a set of beliefs one has about his/her ability to organize and complete a task in order to accomplish a certain task that is crucial for eliciting health behavior change [22]. Self-efficacy is an important predictor to exercise adherence, with numerous trials demonstrating the significant association between improvements in self-efficacy and exercise adherence [23]. Together, these tasks provide the opportunity for the user to modify current behavior in order to meet one’s goals. As such, most mHealth apps have the capacity to allow the user to self-regulate behavior based on past experience and future goals, if guided appropriately. Results of the HEART trial support the continued use of SMS texts to increase exercise engagement through a significant main effect for leisure time physical activity in those receiving the SMS theory-based texts, which was mediated by task self-efficacy.

### Importance of Self-Monitoring

The success of self-regulation is partly dependent on the fidelity, consistency, and timeliness of self-monitoring [24]. Given the instantaneous nature of real-time feedback that mHealth apps can provide, self-monitoring may be carried out promptly and accurately with minimal inconvenience for the individual. However, the process of self-monitoring is not simply an audit of one’s performance [20], and the act of self-monitoring alone is not likely to help an individual self-regulate. Further investment must be taken by looking at past exercise patterns and recognizing barriers. This provision of feedback can provide the individual with an opportunity to evaluate behavior when necessary to remain in line with one’s goal.

### Tailored Feedback

While active engagement in self-monitoring and self-regulation are essential for the maintenance of health-related behavior, it is also imperative to provide individuals with personalized feedback on their behavior [25]. Personalized interventions have been demonstrated to be more effective than nonpersonalized interventions at changing health behavior [25]; however, few interventions utilize this technique. Likewise, a systematic review of SMS-based behavior change interventions confirmed the effectiveness of tailored SMS messages for promoting health behavior change (see literature review by Fjeldsoe et al [25]). In the context of mHealth apps, a personalized intervention would allow a health professional to provide tailored feedback to an individual user in a time-efficient manner.

### This Study

This pilot study sought to examine the utility of a theory-based exercise self-monitoring app for increasing independent exercise adherence over 8 weeks. It was hypothesized that the use of this app would result in (1) more frequent exercise bouts, (2) more frequent self-monitoring, (3) higher perceived self-management of exercise behavior, and (4) higher self-efficacy to self-monitor exercise behavior in comparison to individuals not using the app.

## Methods

### Overview

The study was approved by the University of British Columbia: Okanagan Research Ethics Board. A randomized experimental pilot study design was utilized with participants being randomly selected to one of two conditions—the experimental, app-use condition, or the control, no-app condition. Those randomized to the experimental condition used the app for the 8-week investigation, whereas during the same time period, those randomized to the control condition did not have access to the app. The research assistant met with all participants at both baseline and post-testing time points.

### Participants

**Figure 1:**
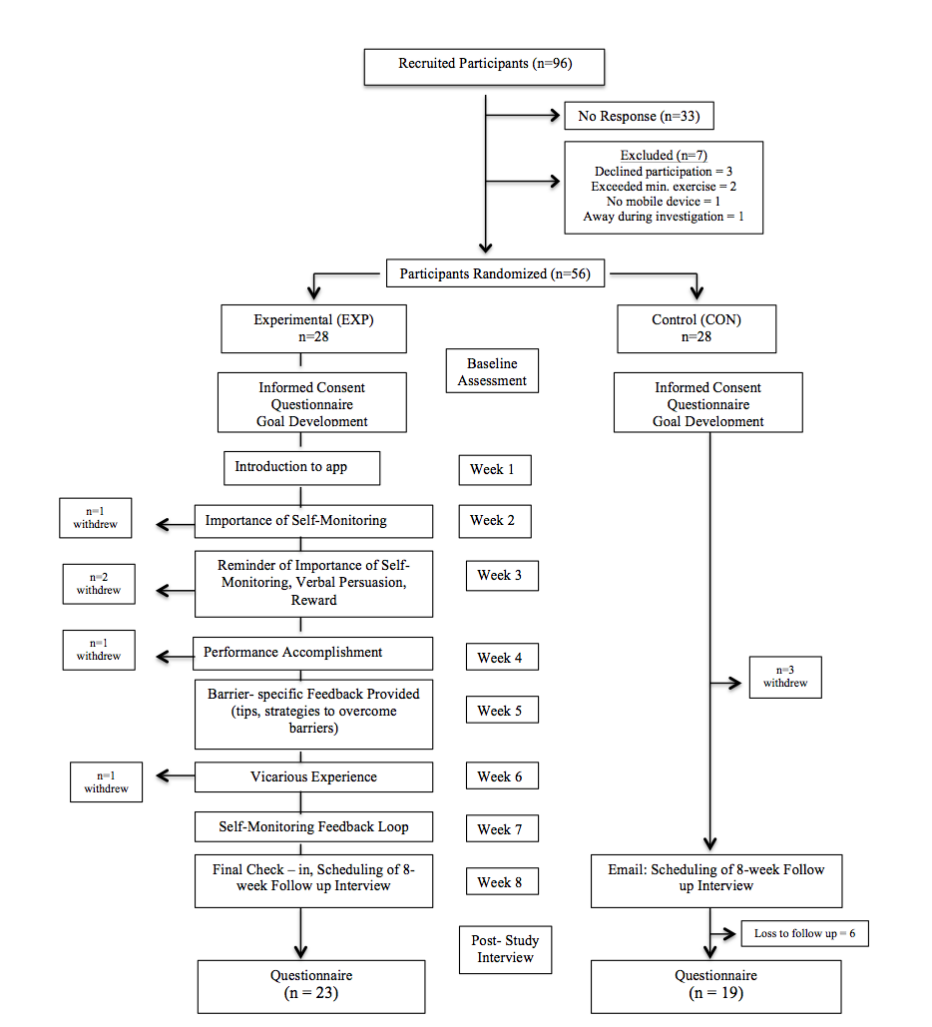
Participant flow.

Participants were recruited from a local YMCA fitness facility by means of announcements in fitness classes, posters located throughout the facility, and an information booth in the lobby. In addition, front desk YMCA staff members were instructed to inform individuals about the study opportunity. Eligible participants were current facility members aged 19-70 years, with access to a mobile device. A total of 94 individuals expressed interest in participating. Following initial screening via email, 56 members were deemed eligible (see Figure 1 for detailed information regarding eligibility); they were then randomized through a computer random numbers-generated table to either the experimental condition, which received the app for 8 weeks (18/28, 64% female), or to the control condition, which did not receive the app (20/28, 71% female). While no specific exercise criteria was set, in meeting with the individual participants it became clear that 2 individuals out of 56 (4%) were excessively active and were engaging in competitive athletic events. These 2 individuals were designated not eligible for participation.

### Procedures

#### Overview

Eligible participants provided written consent and subsequently completed baseline questionnaires. All participants then engaged in a goal-setting discussion using the *specific*, *measureable*, *attainable*, *relevant*, and *time-bound* (SMART) goal-setting framework to self-set a weekly exercise frequency goal (eg, I will visit the gym 3 days this week) for the 8-week study duration.

#### Experimental Condition Protocol

Each participant’s profile was created on the app within 24 hours, at which time he/she was prompted by a text message to sign in and begin monitoring exercise behavior. Participants were encouraged to monitor exercise behavior on a daily basis (ie, record exercise into the app), regardless of whether purposeful exercise was planned or completed that day—from here on referred to as *check-in*. Planned nonexercise days were personalized within the app based on planned bouts of exercise for each week (ie, if an individual’s goal was to exercise three times per week, that participant’s program included 4 *rest*). Participants in the experimental condition were reminded via text message to check in to the app if they had not checked in by 9:00 p.m., regardless of whether they exercised or not that day.

At the beginning of each week, participants were sent a message based on social cognitive theory. Messages ranged from 65 to 135 words in length, and were delivered via the app messaging system, to which users were alerted via a text message. These theory-based messages targeted the components of self-monitoring, verbal persuasion, performance accomplishment, and vicarious experience (see Table 1).

In the event of three consecutive missed check-ins, app users were contacted by the research assistant via SMS text message. If this progressed to four consecutive missed check-ins, the research assistant phoned the participant to discuss any difficulties encountered.

#### Control Condition Protocol

Following goal development, participants in the control condition were encouraged to implement their newly developed goals over the following 8 weeks. Control condition participants did not receive any support from the research assistant throughout the 8-week duration of the study.

#### Follow-Up Protocol for All Participants

At the beginning of week 8, participants in both conditions were contacted via email to schedule a 30-minute follow-up interview for the following week. During this interview, participants completed the poststudy questionnaire.

### Measures

#### Demographics

Participants were asked to provide basic demographic information, including year of birth (see Table 3), height, weight—presented as mean body mass index (BMI) in Table 3 —sex, highest level of education completed, and current occupational status (see Table 4 in Results).

On the fourth day of each week, a second message was sent through the app, delivering tailored feedback and support based on the participant’s personal performance that week. Daily performance was measured on a 5-star rating system (ie, 5 stars represented complete goal achievement for that day and 3 stars represented partial goal achievement for that day). An additional message was sent through the app if a participant failed to check in to the app on 2 consecutive days (see Table 2).

**Table 1:** Overview of weekly theory-based messages to participants.

Week	Message type	Theoretical content
1	Introduction (establishing rapport)	Hi (insert name)! My name is (insert counselor’s name) and I am your virtual exercise counselor. I can’t wait to see the progress you make as you monitor and modify your behavior. I know you are super motivated and ready to kick start your exercise so let’s get you moving! Check in each day to report your activity and keep an eye on your message center. I’ll be checking in frequently to see how you’re doing. Feel free to contact me if you have any questions or concerns.☺
2	Importance of self-monitoring	Hey (insert name)! Just wanted to say you’re doing a great job! You’re already 1 week into using this app and you have tracked your behavior each day! Keeping track of your behavior allows you, and me, to see what a great job you are doing, and helps remind you of your goals. It can also show us where improvements are needed or whether there are any patterns that are problematic. Some people say that keeping track of what exercise they have done is the hardest part—you are excelling in this and this is what will keep you accountable to your personal goals! Keep checking in everyday and let’s rock this!
3	Reminder of importance of self-monitoring, use of verbal persuasion, and self-set rewards	Week 2 down and look at how far you have come! You have now been tracking your exercise behavior for 2 weeks. Keep in mind that self-monitoring is the key to making lasting behavior changes. With this app, tracking your behavior is easy and you are showing yourself that you can do it. You are holding yourself to those goals that you care about so much—doesn’t it feel great? Now is a great time to plan a reward for yourself. Keep up the great work performing and monitoring your exercise—you can do it!
4	Performance accomplishment	(insert name)—Wow look at all you’ve done so far! Take a look at your progress graph—all of those green bars you’ve accumulated are proof that you are well on your way to achieving your goals! You really are using this app to its full potential and you are in control of your exercise. You are doing fantastically—keep up this great momentum.
5	Feedback tailored to participant’s goals/overcoming perceived barriers	Example: Barrier = family time As the weather gets warmer, take the family out on the weekend to kick around a soccer ball, ride bikes, or walk to the park and toss a Frisbee.
6	Establishing vicarious experience	Did you know that you are not the only one going through this program right now? There are 40 other facility members just like you that are monitoring their exercise, trying to achieve their personal exercise goals, and using this app to help them reach those goals. These individuals have been recording their bouts of exercise on the app, and have been overcoming their exercise barriers. So far, the app has been keeping people honest and committed to their exercise goals.
7	Self-monitoring feedback loop	It’s week 7! You’re doing such a fantastic job taking charge of your own exercise regime by consistently monitoring your behavior and achieving positive scores each day. Now is a good time to look back and search for patterns of when you typically find it most difficult to stick with your exercise regimen. This can give you clues on how to circumvent those less-than-optimal motivational days. Notice weekends are your weak point? Be sure to get all your exercise in during the week and take the weekends off on purpose! Finding AM workouts unbearable? Modify your nighttime routine so that getting up and out the door isn’t so hard. By seeking out problematic trends, you can revise your plans and will be more likely to succeed.
8	Final check-in	Today marks the final week of goal tracking for the study. Think about where you started and look at where you are now, the physical and mental barriers that you have been able to break down, and all about what you have learned about yourself. You are in control of your behavior and you are in the habit of self-monitoring. You should feel proud of the progress you’ve made. Now use this feeling to rock your last week of workouts and use this as you move forward. Great job!

**Table 2:** Exercise counselor intervention timeline.

Achievement	Intervention message
Complete daily goal achievement	*First occurrence* Great job yesterday! You successfully completed all your set goals and rocked it! Keep up the great work. *Continuous achievement* (Do not repeat until 1 week after first congratulatory message) Wow! You continually rock it! You’re a rock star! Keep up the good work! You deserve a gold star. Keep rocking it!
Partial daily goal achievement	You’ve had some challenges but you did it! Good job for facing your barriers and getting out there. Keep up the good work.
Partial goal achievement on multiple days	Good job for checking into the app. I know that can be hard when barriers present themselves but you are aware of what is not working. I know you are able to reach those goals you set out to achieve. You can do this!
Missed check-in for 2+ days	Hey (insert name)! Just checking in to see how it’s going! 2 days have gone by since you last checked in to the system. Every day is a new one so let’s get you back on track and start monitoring that exercise! The hardest part is checking in and keeping track of what you are doing. If you have any questions or concerns please do not hesitate to contact me.

**Table 3:** Mean age and body mass index of participants.

Participant characteristics	Control group	Experimental group
Age (years), mean (SD)	41.53 (10.90)	37.45 (14.13)
BMI^a^ (kg/m^2^), mean (SD)	25.87 (3.60)	28.24 (6.50)

^a^BMI: body mass index.

#### Self-Reported Exercise

Purposeful exercise behavior was measured using the Godin Leisure Time Exercise Questionnaire (GLTEQ) [26] at baseline and postintervention (ie, 8 weeks). Participants were asked to report the frequency in which they engaged in moderate (eg, fast walking) and strenuous (eg, jogging) activity during their free time over the past 7 days. The GLTEQ asks participants to report exercise bouts of 30 minutes or more, as recommended by Amireault and Godin [27]. Consistent with past literature [27,28], moderate and strenuous bouts of exercise were summed together for analyses.

#### Self-Monitoring of Exercise Behavior

Assessing the frequency of self-monitoring throughout the 8-week study duration required condition-specific measures. At baseline, all participants’ self-reported the frequency of their self-monitoring exercise behavior over the past 7 days. Postintervention, frequency of self-monitoring exercise behavior among the app users (ie, experimental condition) was assessed using the total number of completed app check-ins, averaged over the 8-week duration of the study. Participants in the control condition were asked to provide an average weekly self-monitoring frequency over the previous 8 weeks.

#### Self-Management of Exercise Behavior

Self-management of exercise was measured using six items from Hallam and Petosa’s [29] measure of self-regulation. Responses were rated on a 5-point Likert scale ranging from 1 (*strongly disagree*) to 5 (*strongly agree*). Relevant items selected assessed plans to participate in exercise (ie, It is difficult for me to find opportunities to participate in exercise) and confidence to self-manage time (ie, I am able to find or make time to participate in exercise). These items were modified from the original measure by replacing the reference from “my condition” to “exercise.” Cronbach alpha values at pre- and postintervention (alpha>.8) suggest a reliable relationship for analysis [30,31].

#### Self-Efficacy to Self-Monitor Exercise

Self-efficacy to self-monitor (SESM) exercise was assessed using three items. Participants rated their confidence to self-monitor exercise bouts on an 11-point Likert scale ranging from 0% (*not at all confident*) to 100% (*extremely confident*). The items assessed participants’ confidence to record exercise (ie, record your exercise bouts), track and adjust behavior (ie, keep track of how many times you exercise and adjust your behavior accordingly), and manage their daily schedule to allow for exercise (ie, manage your daily schedule to allow time for participation in exercise) over the next 7 days. The three items were averaged to reflect an overall SESM score. In this study, scores derived from this instrument demonstrated acceptable levels of reliability (Cronbach alpha>.7) at both pre- and postintervention [30,31].

### Analytic Plan

Data were analyzed using SPSS Statistics version 21 (IBM Corp). A series of independent *t* chi-square tests were conducted to examine equivalency between conditions on all demographic, dependent, and independent variables at baseline. Repeated-measures analyses of variance (ANOVA) were used to test the hypotheses that exercise bouts, self-monitoring exercise, self-efficacy to self-monitor, and self-management of exercise will be greater in the experimental condition as compared to the control condition following the 8-week study period. All effects are reported as significant at *P*<.05. Effect size estimates were calculated using partial eta squared (partial η^2^) for ANOVA and Cohen’s d for *t*. Effect size derived from partial η^2^was interpreted as small (0.01), medium (0.06), and large (0.14) in accordance with conventional practices within the social sciences [32,33]. Likewise, effect sizes derived from Cohen’s d were interpreted as small (0.20), medium (0.50), and large (0.80) [33,34]. Differences between conditions and time × condition interactions yielding medium-to-large effect sizes were further explored with *t*.

## Results

### Demographics

A total of 56 adults—mean age 40 years (SD 13); mean BMI 26.8 kg/m^2^ (SD 5.3)—participated in this study, with 36% of participants having achieved a university level degree or higher, and 50% working either part time or full time. A total of 28 individuals out of 56 (50%) were randomized to the experimental condition—mean age 38 years (SD 14); mean BMI 28.2 kg/m^2^ (SD 6.5)—and 28 out of 56 (50%) were randomized to the control condition—mean age 42 years (SD 11); mean BMI 25.9 kg/m^2^ (SD 3.6). In total, 41 out of 56 participants (73%) provided follow-up data 8-weeks postintervention (see Table 4 for demographic information and Figure 1 for participant flowchart).

There were no statistical differences in demographic, dependent, or independent variables between conditions at baseline with the exception of current exercise self-monitoring behavior. At baseline, participants in the control condition (mean 1.89, SD 2.28) reported a higher frequency of self-monitoring in the past 7 days than participants in the experimental condition (mean 0.52, SD 1.61; *t*_37_=-2.34, *P*=.02, d=0.69). There was no statistical difference in dropout rate between conditions (χ^2^_1_= 1.2, *P*=.28).

### Self-Reported Exercise

A repeated-measures ANOVA examining self-reported exercise frequency revealed no main effect for time (*F*_1,38_=2721, *P*=.11, partial η^2^=.067) or condition (*F*_1,38_= 2.45, *P*=.13, partial η^2^=.061). The time (pre, post) × condition (experimental, control) interaction, although not meeting statistical significance, yielded a medium-to-large effect size (*F*_1,38_= 3.87, *P*=.06, partial η^2^=.092). Exploratory post hoc analysis revealed a significant difference between conditions, such that those in the experimental condition (mean 7.24, SD 3.40) were engaging in significantly more bouts of exercise per week than those in the control condition (mean 4.74, SD 3.70; *t*_38_= 2.23, *P*=.03, d=0.70) (see Table 5).

### Self-Monitoring of Exercise Behavior

A repeated-measures ANOVA examining self-monitoring frequency showed a main effect for time (*F*_1,33_= 59.55, *P*<.001, partial η^2^=.643) and condition (*F*_1,33_= 15.38, *P*<.001, partial η^2^=.318). These main effects were superseded with a significant time (pre, post) × condition (experimental, control) interaction (*F*_1,33_= 49.39, *P*<.001, partial η^2^=.599). Post hoc analysis revealed a significant difference between conditions at postintervention, such that those in the experimental condition (mean 6.00, SD 0.93) were engaging in a significantly higher frequency of self-monitoring compared to the control condition (mean 1.95, SD 2.58; *t*_40_= 6.88, *P*<.001, d=2.10) (see Table 5).

### Self-Management of Exercise Behavior

A repeated-measures ANOVA examining self-management of exercise behavior revealed no main effect for time (*F*_1,38_= 1.91, *P*=.18, partial η^2^=.048) or condition (*F*_1,38_=.408, *P*=.53, partial η^2^=.011). The time (pre, post) × condition (experimental, control) interaction was not significant (*F*_1,38_=.039, *P*=.85, partial η^2^=.001) (see Table 6).

**Table 4:** Participant demographic characteristics.

Participant characteristics			Control group (n=28)^a^, n (%)		Experimental group (n=28)^b^, n (%)
Sex (female)			16 (57)		16 (57)
Education					
	Less than high school		0 (0)		0 (0)
	High school		5 (18)		5 (18)
	Apprenticeship, trades, or diploma		2 (7)		3 (11)
	College		4 (14)		4 (14)
	University diploma or degree		7(25)		7 (25)
	Postgraduate degree		0 (0)		2 (7)
Occupation					
	Working full time		8 (29)		9 (32)
	Working part time		4 (14)		3 (11)
	Working occasionally/contract work		1 (4)		1 (4)
	Student		0 (0)		4 (14)
	Retired		1 (4)		2 (7)
	Other		4 (14)		2 (7)

^a^Only 18 out of 28 control group participants completed these measures.

^b^Only 21 out of 28 experimental group participants completed these measures.

**Table 5:** Self-reported exercise and self-monitoring frequency.

Category	Control group, average frequency/7 days (SD)		Experimental group, average frequency/7 days (SD)	
	Preintervention	Postintervention	Preintervention	Postintervention
Exercise engagement	4.92 (2.91)	4.74 (3.70)	5.14 (3.14)	7.24 (3.40)
Self-monitoring frequency	1.89 (2.28)	1.95 (2.58)	0.52 (1.61)	6.00 (0.93)

**Table 6:** Self-management of exercise behavior.

Category	Control group, average frequency/7 days (SD)		Experimental group, average frequency/7 days (SD)	
	Preintervention	Postintervention	Preintervention	Postintervention
Self-management	3.34 (0.82)	3.16 (0.32)	3.42 (0.86)	3.29 (0.29)

**Table 7:** Self-efficacy to self-monitor exercise.

Category	Control group, average perceived % self-efficacy to self-monitor		Experimental group, average perceived % self-efficacy to self-monitor	
	Preintervention	Postintervention	Preintervention	Postintervention
Self-efficacy to self-monitor	80.70	81.05	83.71	84.70

### Self-Efficacy to Self-Monitor Exercise

A repeated-measures ANOVA examining SESM was conducted, revealing no main effect for time (*F*_1,39_=.092, *P*=.76, partial η^2^=.002) or condition (*F*_1,39_=.665, *P*=.42, partial η^2^=.017). Further, the time (pre, post) × condition (experimental, control) interaction was not significant (*F*_1,39_=.021, *P*=.89, partial η^2^=.001) (see Table 7).

## Discussion

### Principal Findings

This preliminary pilot study investigated the utility of a theory-based self-monitoring app for improving exercise adherence. To our knowledge, this study is the first to integrate behavior change theory in an app, using personalized goals and interaction with a virtual exercise counselor for the promotion of exercise behavior. Findings provide preliminary evidence that, after 8 weeks, individuals with access to such an app engage in a higher frequency of exercise behavior in comparison to individuals who did not have the app. Specifically, app users reported engaging in 7.2 bouts of exercise per week after 2 months, whereas individuals without use of the app reported engaging in 4.7 bouts of exercise per week at this time period. Although this did not reach statistical significance (*P*=.06), this difference between conditions on exercise behavior represents a medium-to-large effect size (partial η^2^=.092), findings that are similar to those reported in other mHealth trials [35,36]. Possible reasons for these positive findings is that mHealth apps allow feedback messages to be sent in a time-sensitive manner, designed around the individual user to further facilitate communication [36]. Use of an app is found to be a simple self-monitoring tool, serving as a means of encouragement and motivation. When combined with feedback, both visual and verbal, the use of an app encourages users to work toward their activity goals [35].

Findings from this pilot study also provide partial support for our secondary hypothesis that use of a theory-based self-monitoring app will result in a higher frequency of self-monitoring in comparison to individuals without access to an app. From baseline to 8 weeks later, app users’ self-monitoring frequency increased from less than one event per week, to an average of six self-monitoring events per week. Self-reported self-monitoring of exercise behavior was unchanged from baseline to post-testing in the control condition (see Table 5). Such an increase in self-monitoring can be partially accredited to the electronic nature of our mHealth app. Electronic diaries facilitate (1) the instantaneous transfer of data between user and counselor or health care provider [37] and (2) have been shown to be associated with higher rates of adherence when compared to traditional self-monitoring via paper and pen diaries [38].

Despite increases in both exercise and self-monitoring behavior, our hypotheses that use of the app would result in an improved self-management of exercise behavior, or self-efficacy to self-monitor exercise behavior was not supported. Use of the app did not result in a significant effect on self-management of exercise from pre- to post-testing time points (see Table 6) or self-efficacy to self-monitor exercise behavior (see Table 7). In regard to the control condition, participants showed no significant change in self-management of exercise or self-efficacy to self-monitor across time points. In light of our nonsignificant findings for self-management of exercise behavior, further evaluation is warranted to understand the manner in which our intervention targeted self-regulatory principles. The purpose of this intervention was to increase the practice of self-monitoring as a key component of self-regulation, and not overcoming exercise barriers. As such, one plausible explanation for the failure to change perceived self-management of exercise behavior in the experimental condition is that this construct was not adequately addressed in the intervention content.

It is also possible the items used to measure self-regulation did not adequately measure the construct within the context of exercise. Although Hallam and Petosa’s [29] measure is highly regarded with respect to self-management of a general health condition, it is plausible that the measure was not context appropriate to measure change in exercise behavior. Further, as can be seen by examination of the means for self-efficacy to self-monitor, a possible ceiling effect may have occurred during this intervention, with baseline self-efficacy scores of over 80% being reported by both conditions. Interestingly, Hallam and Petosa [29] also suggested a problematic ceiling effect in their 2004 study integrating social cognitive theory in a work-site intervention. The purpose of this study was to directly impact self-monitoring through tangible use of an app. However, given the widespread use of apps, one possible explanation for the observed ceiling effect is that all participants were familiar with the act of self-monitoring through other generic apps (eg, tracking work or time spent on social media) prior to the investigation, and therefore their belief (ie, self-efficacy) to self-monitor was not significantly impacted through the intervention material.

### Strengths and Limitations

The integration of theory into the development of a self-monitoring app was the primary strength of this pilot study. To date, principles from theories of health behavior have been used sparingly within mHealth apps [12], despite evidence to suggest the integration of theory (eg, social cognitive theory) lends support to behavior change [18]. In this study, app users received a social cognitive theory-grounded message once per week over 8 weeks. Such an automated strategy could feasibly be incorporated into many existing mHealth apps. Further, each app user set a personalized 8-week goal, allowing for tailored feedback from a virtual exercise counselor. Lack of tailored feedback has been a limitation in previous trials [35]. The current trial was able to integrate the use of tailored, real-time feedback in a nonburdensome manner, facilitated by one exercise counselor. Daily review of users’ self-monitoring was made manageable due to the electronic nature of the app, taking approximately one minute per day per participant to review and respond to users’ questions and comments. As the system is developed with a pre-existing bank of messages for weekly, theory-based content (see Table 1) and an established timeline of when to intervene (see Table 2), contact between the exercise counselor and user is as simple as choosing a situation-specific message and further specifying details based on the individual.

This study is not without limitations. Given the nature of this investigation acting as a pilot trial, and in working with an entrepreneurial developer, a power calculation was not performed out of logistics in working with our industry partner and recruitment time constraints. Recruitment for this study was limited to one fitness facility due to restrictions placed by the app industry partner, resulting in limited power to detect group differences as well as an inability to conduct more complex analyses to better understand why potential differences existed (eg, mediation and multiple mediation). These findings may not be generalizable to individuals who are not able to afford fitness facility memberships; however, it should be noted that the facility utilized in this study offers subsidized memberships based on gross income. Given the general recruitment criteria (ie, 19-70 years of age, access to a mobile phone device), the heterogeneity of our sample may have weakened our ability to draw concise conclusions and apply them to the general population as not all participants were new to exercise and may have had prior experience with mHealth app technology. Self-monitoring behavior was the secondary focus of this intervention. While app users’ self-monitoring frequency was calculated using data from the app, the self-monitoring frequency of participants in the control condition (ie, no app) was based on self-report data, as the control aspect of this study design prohibited measurement via an app of these participants. The use of self-report data is inherent to recall bias [36], potentially resulting in unreliable results in the comparison of conditions. Lastly, this study looked at changes in exercise behavior over the duration of an 8-week intervention. As 40-65% of new exercisers drop out within the first 6 months [4] of a new program, an extended trial of the app is warranted to draw conclusions on long-term efficacy.

### Future Directions

Wearable devices (eg, fitness trackers, pedometers, and accelerometers) have become sophisticated, with continued development of technology bringing credibility to such devices. Continued research on the development of mHealth devices could help to establish users' trust in the integration of technology (eg, mobile phone apps and wearable devices) to monitor health behaviors. Overall, an enhanced trust in the use of technology could have a meaningful effect on the ability of a device to impact the health of the public in general, as well as specialized populations [39]. Future studies should look to the integration of a true control group with access to a general health-related self-monitoring app, and objectively measure self-monitoring in the control condition, to provide further understanding of the mechanism under which users are affected by mHealth technology.

### Conclusions

A total of 8 weeks of mHealth app use resulted in increased exercise and self-monitoring behavior, providing some support for the use of a self-monitoring app to increase adherence to exercise and self-monitoring of exercise behavior. This study protocol also demonstrates the feasibility of incorporating theory-based messages into existing mHealth apps, although the inclusion of such content did not lead to anticipated changes in self-efficacy to self-monitor or self-management of exercise behavior. Multiple inoculations of theory-based messages may be needed for sizable changes to be made in these constructs. Future research is warranted to understand the long-term efficacy of an mHealth app and its effect on exercise and self-monitoring behavior.

## Abbreviations

ANOVA: analysis of variance
BMI: body mass index
GLTEQ: Godin Leisure Time Exercise Questionnaire
HEART: Heart Exercise And Remote Technologies
SESM: self-efficacy to self-monitor
SMART: specific, measureable, attainable, relevant, and time-bound
SMS: short message service

## References

[1] Vina J, Sanchis-Gomar F, Martinez-Bello V, Gomez-Cabrera MC (2012). Exercise acts as a drug: The pharmacological benefits of exercise. Br J Pharmacol.

[2] Tremblay MS, Warburton DE, Janssen I, Paterson DH, Latimer AE, Rhodes RE, Kho ME, Hicks A, Leblanc AG, Zehr L, Murumets K, Duggan M (2011). New Canadian physical activity guidelines. Appl Physiol Nutr Metab.

[3] Canadian Society for Exercise Physiology (2012). Canadian Physical Activity Guidelines and Canadian Sedentary Behaviour Guidelines.

[4] Annesi J (2003). Effects of a cognitive behavioral treatment package on exercise attendance and drop out in fitness centers. Eur J Sport Sci.

[5] Dishman R (1991). Increasing and maintaining exercise and physical activity. Behav Ther.

[6] Colley R, Garriguet D, Janssen I, Craig C, Clarke J, Tremblay M (2011). Physical activity of Canadian children and youth: Accelerometer results from the 2007 to 2009 Canadian Health Measures Survey. Health Rep.

[7] Al Ayubi SU, Parmanto B, Branch R, Ding D (2014). A persuasive and social mHealth application for physical activity: A usability and feasibility study. JMIR Mhealth Uhealth.

[8] Leslie E, Sparling P, Owen N (2001). University campus settings and the promotion of physical activity in young adults: Lessons from research in Australia and the USA. Health Educ.

[9] Hall AK, Cole-Lewis H, Bernhardt JM (2015). Mobile text messaging for health: A systematic review of reviews. Annu Rev Public Health.

[10] Rainie L, Fox S (2012). Pew Research Center.

[11] Cowan LT, Van Wagenen SA, Brown BA, Hedin RJ, Seino-Stephan Y, Hall PC, West JH (2013). Apps of steel: Are exercise apps providing consumers with realistic expectations?: A content analysis of exercise apps for presence of behavior change theory. Health Educ Behav.

[12] Patel MS, Asch DA, Volpp KG (2015). Wearable devices as facilitators, not drivers, of health behavior change. JAMA.

[13] Rabin C, Bock B (2011). Desired features of smartphone applications promoting physical activity. Telemed J E Health.

[14] Direito A, Dale LP, Shields E, Dobson R, Whittaker R, Maddison R (2014). Do physical activity and dietary smartphone applications incorporate evidence-based behaviour change techniques?. BMC Public Health.

[15] Abroms LC, Padmanabhan N, Thaweethai L, Phillips T (2011). iPhone apps for smoking cessation: A content analysis. Am J Prev Med.

[16] Doshi A, Patrick K, Sallis JF, Calfas K (2003). Evaluation of physical activity web sites for use of behavior change theories. Ann Behav Med.

[17] Riley WT, Rivera DE, Atienza AA, Nilsen W, Allison SM, Mermelstein R (2011). Health behavior models in the age of mobile interventions: Are our theories up to the task?. Transl Behav Med.

[18] Maddison R, Pfaeffli L, Stewart R, Kerr A, Jiang Y, Rawstorn J, Carter K, Whittaker R (2014). The HEART mobile phone trial: The partial mediating effects of self-efficacy on physical activity among cardiac patients. Front Public Health.

[19] Bandura A (1986). Social Foundations of Thought and Action: A Social Cognitive Theory.

[20] Bandura A (1991). Social cognitive theory of self-regulation. Organ Behav Hum Decis Process.

[21] Sniehotta F, Scholz U, Schwarzer R (2005). Bridging the intention-behaviour gap: Planning, self-efficacy, and action control in the adoption and maintenance of physical exercise. Psychol Health.

[22] Bandura A (1977). Self-efficacy: Toward a unifying theory of behavioral change. Psychol Rev.

[23] Garcia A, King A (1991). Predicting long-term adherence to aerobic exercise: A comparison of two models. J Sport Exerc Psychol.

[24] Burke LE, Wang J, Sevick MA (2011). Self-monitoring in weight loss: A systematic review of the literature. J Am Diet Assoc.

[25] Fjeldsoe BS, Marshall AL, Miller YD (2009). Behavior change interventions delivered by mobile telephone short-message service. Am J Prev Med.

[26] Godin G, Shephard R (1997). Godin leisure-time exercise questionnaire. Med Sci Sports Exerc.

[27] Amireault S, Godin G (2015). The Godin-Shephard leisure-time physical activity questionnaire: Validity evidence supporting its use for classifying healthy adults into active and insufficiently active categories. Percept Mot Skills.

[28] Parrott M, Tennant L, Olejnik S, Poudevigne M (2008). Theory of planned behavior: Implications for an email-based physical activity intervention. Psychol Sport Exerc.

[29] Hallam JS, Petosa R (2004). The long-term impact of a four-session work-site intervention on selected social cognitive theory variables linked to adult exercise adherence. Health Educ Behav.

[30] Gliem J, Gliem R (2003). Calculating, interpreting, and reporting Cronbach's alpha reliability coefficient for Likert-type scales. Proceedings of the Midwest Research-to-Practice Conference in Adult, Continuing, and Community Education.

[31] Slavec A, Drnovšek M (2012). A perspective on scale development in entrepreneurship research. Econ Bus Rev.

[32] (2016). MRC Cognition and Brain Sciences Unit.

[33] Cohen J (1988). Statistical Power Analysis for the Behavioral Sciences. 2nd edition.

[34] Fritz CO, Morris PE, Richler JJ (2012). Effect size estimates: Current use, calculations, and interpretation. J Exp Psychol Gen.

[35] Fukuoka Y, Lindgren T, Jong S (2012). Qualitative exploration of the acceptability of a mobile phone and pedometer-based physical activity program in a diverse sample of sedentary women. Public Health Nurs.

[36] Maddison R, Pfaeffli L, Whittaker R, Stewart R, Kerr A, Jiang Y, Kira G, Leung W, Dalleck L, Carter K, Rawstorn J (2015). A mobile phone intervention increases physical activity in people with cardiovascular disease: Results from the HEART randomized controlled trial. Eur J Prev Cardiol.

[37] Burke LE, Warziski M, Starrett T, Choo J, Music E, Sereika S, Stark S, Sevick MA (2005). Self-monitoring dietary intake: Current and future practices. J Ren Nutr.

[38] Burke LE, Styn MA, Sereika SM, Conroy MB, Ye L, Glanz K, Sevick MA, Ewing LJ (2012). Using mHealth technology to enhance self-monitoring for weight loss: A randomized trial. Am J Prev Med.

[39] Case MA, Burwick HA, Volpp KG, Patel MS (2015). Accuracy of smartphone applications and wearable devices for tracking physical activity data. JAMA.

